# Chemosensitisation by manganese superoxide dismutase inhibition is caspase-9 dependent and involves extracellular signal-regulated kinase 1/2

**DOI:** 10.1038/sj.bjc.6604477

**Published:** 2008-07-01

**Authors:** B H Y Yeung, K Y Wong, M C Lin, C K C Wong, T Mashima, T Tsuruo, A S T Wong

**Affiliations:** 1 School of Biological Sciences, The University of Hong Kong, Hong Kong, P.R. China; 2 Department of Chemistry, The University of Hong Kong, Hong Kong, P.R. China; 3 Department of Biology, Hong Kong Baptist University, Hong Kong, P.R. China; 4 Cancer Chemotherapy Center, Japanese Foundation for Cancer Research, Tokyo, Japan

**Keywords:** MnSOD, caspases, ovarian cancer, apoptosis, chemotherapy

## Abstract

Chemoresistance and therapeutic selectivity are major obstacles to successful chemotherapy of ovarian cancer. Manganese superoxide disumutase (MnSOD) is an important antioxidant enzyme responsible for the elimination of superoxide radicals. We reported here that MnSOD was significantly elevated in ovarian cancer cells and its overexpression was one of the mechanisms that increased resistance to apoptosis in cancer cells. Knockdown of MnSOD by small-interfering RNA (siRNA) led to an increase in superoxide generation and sensitisation of ovarian cancer cells to the two front-line anti-cancer agents doxorubicin and paclitaxel whose action involved free-radical generation. This synergistic effect was not observed in non-transformed ovarian surface epithelial cells. Furthermore, our results revealed that this combination at the cellular level augmented activation of caspase-3 and caspase-9, but not caspase-8, suggesting involvement of an intrinsic apoptotic pathway. Evaluation of signalling pathways showed that MnSOD siRNA enhanced doxorubicin- and paclitaxel-induced phosphorylation of extracellular signal-regulated kinase 1/2. Akt activation was not affected. These results identify a novel chemoresistance mechanism in ovarian cancer, and show that combination of drugs capable of suppressing MnSOD with conventional chemotherapeutic agents may provide a novel strategy with a superior therapeutic index and advantage for the treatment of refractory ovarian cancer.

Ovarian cancer is the fourth or fifth most common cancer in North America and Europe, and it is the leading cause of death from all types of gynaecologic cancer (Jemal *et al*, 2006). The majority of patients present with advanced-stage (stage III or IV) disease and have a poor prognosis. The 5-year survival rate is only 16–28% (Heintz *et al*, 2001; Tingulstad *et al*, 2003). Currently, cytotoxic chemotherapy that kills cancer cells mainly by apoptosis is the standard treatment to prevent ovarian cancer recurrences after maximal cytoreductive surgery or to treat recurrences (Aletti *et al*, 2007). However, commonly used cytotoxic chemotherapeutic agents often have narrow therapeutic indices due to highly non-specific cytotoxicity and undesirable side effects. Furthermore, tumours tend to acquire resistance to cytotoxic chemotherapeutic agents, and in most the disease remain incurable.

The molecular basis of drug resistance is not well understood, although a few speculative mechanisms associated with the enhanced antioxidant capacity of tumour cells have been suggested. Superoxide dismutases (SODs) are important antioxidant enzymes responsible for the elimination of superoxide (O_2_^•−^) radicals. There are three known SODs, which are cytosolic copper/zinc-containing SOD (Cu/Zn-SOD) and the mitochondrial manganese-containing SOD (MnSOD). These SODs catalyse dismutation of O_2_^•−^ into hydrogen peroxide which is then catalysed to O_2_ and H_2_O by glutathione (GSH) peroxidase and catalase (Halliwell and Gutteridge, 1999). As mitochondria respiration is the main source of O_2_^•−^ generation in the cells, MnSOD is of prime importance in maintaining the cellular ROS balance. Genetic knockout studies in mice indicate that MnSOD, but not other SODs, is essential for cell survival (Carlsson *et al*, 1995; Li *et al*, 1995; Reaume *et al*, 1996). Although numerous studies have demonstrated altered antioxidant enzyme activity in a variety of solid tumours, ovarian cancer is unique because generation of ROS appears to be a risk factor for ovarian cancer (Murdoch, 2005). This is a scenario that may not be found with most other malignancies and challenges the view of MnSOD as a tumour suppressor. MnSOD is overexpressed in the majority (70–80%) of ovarian cancer tissues and the level of expression of MnSOD is also positively correlated with more advanced features in ovarian cancer patients, such as tumour grade and disease progression (Ishikawa *et al*, 1990; Hileman *et al*, 2004; Hu *et al*, 2005). Most interesting, MnSOD overexpression has been shown to suppress apoptosis induced by anti-cancer drugs in various cell types (Hirose *et al*, 1993). However, the detailed mechanism underlying MnSOD protection from anti-cancer drug-induced cell death is still largely unknown.

In this study, we show for the first time that overexpression of MnSOD is one of the mechanisms that increases resistance to apoptosis in ovarian cancer cells. We also report a strategy to inhibit MnSOD and increase O_2_^•−^ generation in ovarian cancer cells as a novel mechanism to enhance apoptosis induced by doxorubicin (DOX) and paclitaxel (PTX), the two front-line anti-cancer agents used in ovarian cancer therapy, leading to a preferential killing of the cancer cells. Our results further delineate a novel mechanism for extracellular signal-regulated kinase (ERK)1/2-dependent up-regulation of caspase-9 that contributes to this synergistic effect.

## Materials and methods

### Antibodies and reagents

Polyclonal antibodies against Akt, phospho-Akt, ERK1/2, phospho-ERK1/2, caspase-9, and caspase-3 were purchased from Cell Signaling (Beverly, MA, USA). Manganese superoxide disumutase, Cu/Zn-SOD polyclonal antibodies were obtained from Stressgen (Victoria, BC, Canada). Caspase-8 antibody was from BD Pharmingen (San Diego, CA, USA) and *β*-actin antibody was from Sigma (St Louis, MO, USA). Peroxidase-conjugated secondary antibodies were obtained from Bio-Rad (Hercules, CA, USA). Caspase-8 inhibitor Z-IETD-FMK, caspase-9 inhibitor Z-LEHD-FMK, and pan-caspase inhibitor Z-VAD-FMK were purchased from R&D Systems, Inc. (Minneapolis, MN, USA). Paclitaxel, DOX, and the MEK1 inhibitor PD98059 were obtained from Calbiochem (San Diego, CA, USA). *N*-Acetyl cysteine (NAC) and GSH were purchased from Sigma. The human MnSOD expression plasmid was a gift from Dr Abbadie at the Institute of Biology of Lille, France (Bernard *et al*, 2001). Dominant-negative mutants of human MEK1 (DN-MEK1; Wang *et al*, 2006) and caspase-9 (Mashima *et al*, 2005) were described elsewhere.

### Cell culture and treatments

Human ovarian carcinoma cell lines (CaOV-3, SKOV-3, and OVCAR-3) and human OSE cell lines (IOSE-29, IOSE-80, IOSE-397, and IOSE-398) were generously provided by Dr N Auersperg at University of British Columbia, Vancouver, BC, Canada. These cell lines were maintained in medium 199 : 105 supplemented with 5% FBS, 100 U ml^−1^ penicillin-streptomycin (Invitrogen, Carlsbad, CA, USA) in a humidified atmosphere of 5% CO_2_ at 37°C. Cells were grown to about 80% confluence and then treated with 5 *μ*M DOX or 100 nM PTX for 48 h.

### Stable and transient transfections

Stable transfection was performed with pcDNA3.1 plasmid-containing MnSOD or an empty vector alone, and colonies were selected in 0.4 mg ml^−1^ G418 (Invitrogen). For transient transfection, at 24 h after transfection, the cells were undergone treatments for further 48 h and lysed for analysis. Each experiment was repeated with three independent transfections. All transfections were performed using the Lipofectamine 2000 transfection reagent according to the manufacturer's instructions (Invitrogen).

### siRNA transfection for knockdown of MnSOD

SKOV-3 cells were transfected with 10 nM MnSOD small-interfering RNA (siRNA) oligonucleotides or scrambled oligonucleotides (Dharmacon Research, Lafayette, CO, USA) using siLectFect (Bio-Rad), according to the manufacturer's instructions. After 48 h, transfected cells were harvested for examining the expression of MnSOD protein by western blot analysis, or subjected to drug treatments, and apoptosis assays.

### ROS assay

Intracellular ROS production was measured using the O_2_^•−^-sensitive probe dihydroethidium (Molecular Probes, Eugene, OR, USA) as described in previous reports (Yeung *et al*, 2006). Briefly, cells were labelled with 2 *μ*M of dihydroethidium for 15 min at 37°C, and dye oxidation (increase in fluorescence) was measured using an EPICS Elite flow cytometer (Beckman-Coulter, Miami, FL, USA) with excitation and emission settings of 488 and 605 nm respectively.

### MnSOD enzymatic activity assay

The MnSOD enzyme activity level was determined by a SOD Assay Kit (Cayman Chemical, Ann Arbor, MI, USA) in accordance with the manufacturer's protocols as described. In brief, this kit allows SOD assay by using a highly water soluble tetrazolium salt for detection of O_2_^•−^ radicals generated by xanthine oxidase and hypoxanthine (Liu *et al*, 2005). The specific activity of MnSOD was determined by adding 2 mM KCN to samples to block Cu/Zn-SOD and extracellular SOD activities completely. MnSOD activity was calculated by using the equation obtained from the linear regression of the standard curve substituting the linearised rate for each sample.

### MTT assay

Cell viability was assessed colorimetrically by the mitochondria-dependent reduction of MTT (3-(4,5-dimethyl-thiazol-2-yl)-2,5-diphenyltetrazolium bromide) (Sigma) to formazan. The absorbance was measured with a microplate reader reading at 570 nm with 630 nm as the reference wavelength. Relative cell viability was expressed as the fold change over control cultures.

### Apoptosis analysis

Cells were analysed for apoptosis by TUNEL (terminal deoxynucleotidyl transferase-mediated dUTP nick-end labelling) using a commercially available *In situ* Cell Death Detection kit (Boehringer Mannheim, Mannheim, Germany) to find DNA strand breaks according to the manufacturer's instructions. To detect morphologic changes in the apoptotic process, cell nuclei were stained with 1 *μ*g ml^−1^ 4′,6-diamidino-2-phenylindole (DAPI; Pon *et al*, 2005). Apoptotic cells were identified by chromatin condensation and nuclei fragmentation. The number of TUNEL/DAPI-positive cells was counted in four different fields with at least 500 cells counted in each instance and representative fields were photographed. To confirm the findings, apoptotic cells were also detected by annexin V staining (BD Pharmingen) following the manufacturer's instructions, as described in previous reports (Pon *et al*, 2005; Yeung *et al*, 2006). Briefly, adherent and floating cells were collected by trypsinisation and washed with PBS twice. Cells were re-suspended in annexin V binding buffer containing FITC-conjugated annexin V. Propidium iodide (PI) at 1 *μ*g ml^−1^ was then added, and samples were incubated for 15 min at room temperature in the dark. The percentage of cells with annexin V^+^/PI^−^ was measured using FACS (Becton Dickinson, San Jose, CA, USA). Each set of experiments were performed in replicate and repeated three times.

### Western blot analysis

Protein (50 *μ*g) was loaded onto 12% SDS–PAGE gels, separated, and transferred onto nitrocellulose membranes (Bio-Rad). The membranes were blocked with 3% non-fat milk and incubated with the appropriate primary antibody overnight at 4°C. After washing, the membranes were incubated with the corresponding peroxidase-conjugated secondary antibody for 1 h at room temperature. Protein bands were visualised by enhanced chemiluminescence system (Amersham Biosciences, Piscataway, NJ, USA).

### Statistical analysis

Data were expressed as the mean±s.d. of at least three separate experiments. Statistical analysis was done using one-way analysis of variance followed by Tukey's least significant difference *t*-test for *post hoc* analysis (GraphPad Software, San Diego, CA, USA), and *P*<0.05 was considered significant.

## Results

### MnSOD protein levels in OSE and cancer cell lines

**Figure 1:**
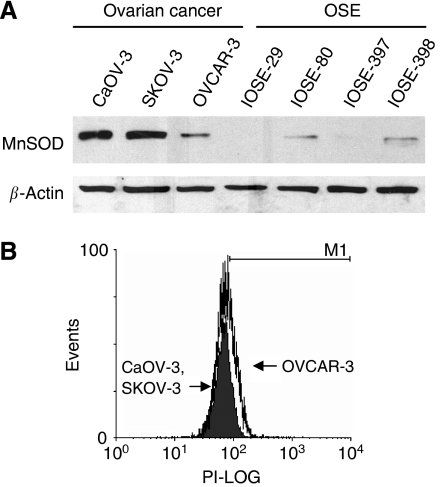
Manganese superoxide disumutase (MnSOD) expression in OSE and ovarian cancer cells. (**A**) Whole-cell lysates from different ovarian cell lines containing equal amounts of protein (50 *μ*g) were loaded in each lane and analysed for expression of MnSOD by western blotting. *β*-Actin was also blotted as a protein-loading control. (**B**) O_2_^•−^ level was measured by flow cytometry analysis using dihydroethidium. Solid histogram, CaOV-3 and SKOV-3 cells; open histogram, OVCAR-3.

It has been shown in earlier studies that the majority of ovarian cancer tissues exhibited higher levels of MnSOD expression as compared with normal or benign ovary tissues (Ishikawa *et al*, 1990; Hileman *et al*, 2004; Hu *et al*, 2005). In the present study, we compared the expression level of MnSOD in three ovarian carcinoma cell lines (CaOV-3, SKOV-3, and OVCAR-3) against immortalised, non-tumourigenic OSE cell lines (IOSE-29, IOSE-80, IOSE-397, and IOSE-398). As shown in Figure 1A, OSE cells exhibited only a weak MnSOD signal. In contrast, all of the ovarian cancer cell lines, with the exception of OVCAR-3, showed substantially higher levels of MnSOD. As MnSOD catalyses the elimination of O_2_^•−^ in the mitochondria where a large portion of cellular ROS is generated, we measured the O_2_^•−^ content by flow cytometry using the ROS-sensitive probe dihydroethidium. Consistently, CaOV-3 and SKOV-3 exhibited relatively lower levels of fluorescence (cellular O_2_^•−^ level) compared with OVCAR-3 (Figure 1B).

### Expression of MnSOD confers resistance to drug-induced apoptosis

To investigate the role of MnSOD in ovarian carcinoma cells, we used gain-of-function and loss-of-function strategies. We selected two cell lines: OVCAR-3, which MnSOD is almost undetectable and sensitive to standard chemotherapy, and SKOV-3, which express high levels of MnSOD and are more resistant to chemotherapy (Gibb *et al*, 1997). The former cell line was transfected with human MnSOD expression vector. SKOV-3 cells were transfected with MnSOD siRNA.

**Figure 2:**
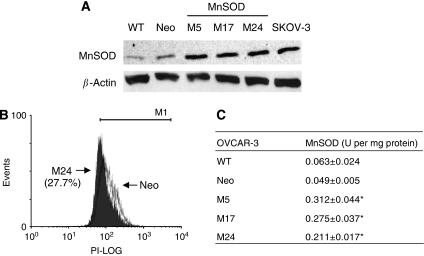
Overexpression of manganese superoxide disumutase (MnSOD) reduces ROS production in OVCAR-3. (**A**) Whole-cell lysates of MnSOD stably transfected clones (M5, M17, and M24), parental (WT), and vector (Neo) control cells were harvested and analysed for expression of MnSOD by western blotting. *β*-Actin serves as a protein-loading control. (**B**) O_2_^•−^ level was measured by flow cytometry analysis using dihydroethidium. Solid histogram, MnSOD-overexpressing clones; open histogram, Neo control. (**C**) MnSOD activity was measured. Values are mean±s.d. of three independent experiments. ^*^P<0.05 *vs* WT and Neo control cells.

We first transfected OVCAR-3 cells with empty vector (pcDNA3.1) or MnSOD-expressing vector. After transfection, cells were cultured in a medium containing 400 *μ*g ml^−1^ of G418. Each colony that grew after G418 selection was picked and expanded. We obtained three independent clones, which expressed MnSOD. Notably, MnSOD was much expressed (3.7- to 3.9-fold) in all of three MnSOD-expressing OVCAR-3 clones (M5, M17, and M24) than in parental (WT) and vector (Neo) control clones (Figure 2A). It is important to note that the levels of MnSOD in these stable cell lines were less than those detected in MnSOD-overexpressing human ovarian carcinoma cell lines, including SKOV-3, indicating that expression was within normal levels (Figure 2A). Analysis (RT–PCR) showed that MnSOD mRNA was also significantly elevated in these stable transfectants (data not shown). Consistent with the western blots, all MnSOD-overexpressing clones showed decrease in ethidium fluorescence, showing that MnSOD is functionally active and able to reduce cellular ROS level (9.4–27.7%) in these cell lines. The representative result from one of them (M24) is shown in Figure 2B. To confirm the levels of MnSOD enzymatic activity, we performed MnSOD activity assays. As shown in Figure 2C, MnSOD-transfected clones had statistically significant increases in MnSOD activity compared with WT, and the activity of Neo was not different from that of WT. The values obtained from the MnSOD activity correlated well with the expression level and the O_2_^•−^ content (*P*<0.05), suggesting that OVCAR-3 constitutively express active MnSOD.

**Figure 3:**
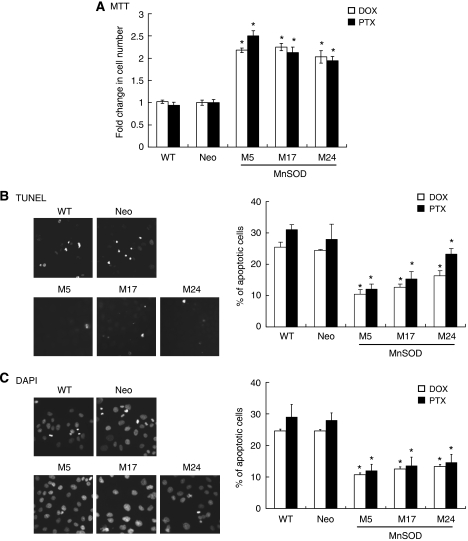
Overexpression of manganese superoxide disumutase (MnSOD) protects cells from doxorubicin (DOX)- and paclitaxel (PTX)-induced apoptosis in OVCAR-3. Manganese superoxide disumutase-overexpressing OVCAR-3 cells were exposed to DOX (5 *μ*M) or PTX (0.1 *μ*M) for 48 h and then harvested for (**A**) 3-(4,5-dimethyl-thiazol-2-yl)-2,5-diphenyltetrazolium bromide (MTT) assay. The absorbance of wells not exposed to treatments was arbitrarily set as 1, and cell growth after treatment was expressed as the fold changes compared with the control. Apoptosis was measured by (**B**) terminal deoxynucleotidyl transferase-mediated dUTP nick-end labelling (TUNEL) and (**C**) 4′,6-diamidino-2-phenylindole (DAPI) staining. Cells stained with DAPI exhibiting condensed, pyknotic, or fragmented nuclei were representative of apoptotic cells. Experiments were repeated three times, and data are shown as mean±s.d. ^*^P<0.05 *vs* WT and Neo control cells.

DOX and PTX are two potent and commonly used chemotherapeutic agents of treating ovarian cancer in the clinic. In addition, both classes of these drugs are able to generate ROS production in cancer cells, and ROS was found to be involved in DOX- and PTX-induced cell death *in vitro* and *in vivo* (Conklin, 2004; Ramanathan *et al*, 2005; Alexandre *et al*, 2006). Therefore, it was of interest to determine whether MnSOD could inhibit their ability to induce apoptosis. The dosages of both DOX and PTX were determined by LD_50_ obtained from MTT assays (Supplementary Figure 1). As shown, MnSOD-overexpressing cells all had greater cell viability than WT and Neo control cells in response to 5 *μ*M DOX or 100 nM PTX (*P*<0.05 *vs* WT and Neo cells; Figure 3A). To determine whether apoptosis accounted for the loss of viability, we first examined the presence of DNA fragmentation by TUNEL. Figure 3B shows that the percentage of TUNEL-positive cells was significantly lower in DOX- or PTX-treated MnSOD-overexpressing cells compared with WT and Neo controls. The ability of MnSOD to suppress apoptosis was examined also by counting DAPI-stained cells with condensed nuclei. Similar to the TUNEL staining experiments, a significant decrease in DOX- or PTX-induced apoptosis was observed in MnSOD-overexpressing clones (Figure 3C). Together, the above results suggest that overexpression of MnSOD rendered OVCAR-3 cells more resistant to apoptosis induction by chemotherapeutic agents.

### Inhibition of MnSOD sensitises ovarian cancer cells to chemotherapy, whereas OSE cells are unaffected

**Figure 4:**
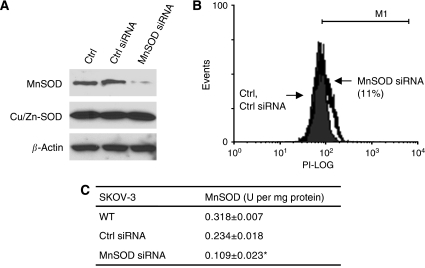
Suppressing manganese superoxide disumutase (MnSOD) expression by small-interfering RNA (siRNA) increases ROS production in SKOV-3. (**A**) Cells transfected with non-specific control (Ctrl siRNA) or MnSOD siRNA were analysed for expression of MnSOD, Cu/Zn-SOD proteins by western blotting using the respective antibodies. *β*-Actin serves as a protein-loading control. (**B**) O_2_^•−^ level was measured by flow cytometry analysis using dihydroethidium. Solid histogram, untransfected and Ctrl siRNA-transfected cells; open histogram, MnSOD siRNA-transfected cells. (**C**) MnSOD activity was measured. Values are mean±s.d. of three independent experiments. ^*^P<0.05 *vs* WT and Neo control cells.

**Figure 5:**
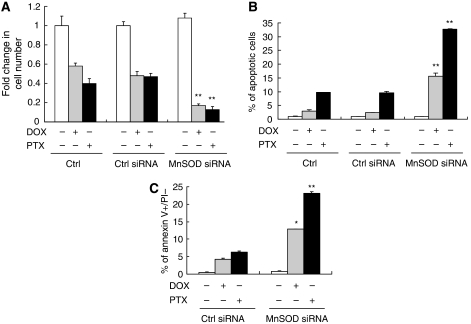
Suppressing manganese superoxide disumutase (MnSOD) expression by small-interfering RNA (siRNA) sensitises SKOV-3 cells to doxorubicin (DOX)- and paclitaxel (PTX)-induced apoptosis. Cells were exposed to DOX (5 *μ*M) or PTX (0.1 *μ*M) for 48 h and then harvested for (**A**) 3-(4,5-dimethyl-thiazol-2-yl)-2,5-diphenyltetrazolium bromide (MTT) assay. The absorbance of wells not exposed to treatments was arbitrarily set as 1, and cell growth after treatment was expressed as the fold changes compared with the control. Apoptosis was measured by (**B**) terminal deoxynucleotidyl transferase-mediated dUTP nick-end labelling (TUNEL) assay and (**C**) by staining with FITC-annexin V and propidium iodide (PI) and analysed by flow cytometry. The per cent of apoptotic cells defined as annexin V^+^/PI^−^ (lower right quadrant) were counted. Experiments were repeated three times, and data are shown as mean±s.d. ^*^P<0.05, ^**^P<0.001 *vs* untransfected and Ctrl siRNA cells.

Subsequently, we used RNA interference to reduce MnSOD expression in SKOV-3 ovarian cancer cell line, which expressed high levels of endogenous MnSOD. Western blot analysis revealed that transfection with MnSOD siRNA specifically suppressed the protein expression of MnSOD and did not affect the expression of Cu/Zn-SOD or *β*-actin. The non-specific (Ctrl) siRNA exhibited no effect on the expression of all these molecules tested (Figure 4A). Moreover, flow cytometric analysis showed that there was an 11% increase in O_2_^•−^ content in cells transfected with MnSOD siRNA, whereas Ctrl siRNA did not cause any significant change in cellular O_2_^•−^ level (Figure 4B). Consistently, MnSOD activity was significantly lower in cells transfected with MnSOD siRNA from the Ctrl siRNA (Figure 4C). Figure 5A shows an ∼70% decrease in cell viability compared with the control. In accordance with this, 5 *μ*M DOX or 100 nM PTX induced 2.5 and 9.6% apoptosis respectively, whereas a combination of DOX or PTX and MnSOD siRNA induced 15.6 and 32.7% apoptosis respectively (*P*<0.001 *vs* untransfected control and Ctrl siRNA; Figure 5B). Untreated and MnSOD siRNA-transfected cells had a very minimal number of apoptotic cells (Figure 5B). We also examined the reactivity to annexin V-FITC in conjunction with PI to detect exposure of dislocated phosphatidylserine to the external face of the plasma membrane, a process regarded as a marker of apoptosis. In view of the cells that are positive for both annexin V and PI may also be cells that are undergoing necrosis, only the percentage of early apoptotic cells (annexin V positive and PI negative) was quantified from three individual experiments and shown in Figure 5C. MnSOD siRNA increased the fraction of apoptotic cells to 12.6 and 23.1% respectively. This magnitude of change was comparable with the fold induction detected by TUNEL and DAPI assays. The slight variation using different techniques can be accounted for by their different sensitivity and detection of specific markers in the apoptotic pathway. Together, these results indicate that DOX or PTX, when used in combination with MnSOD siRNA, induce more efficient (3.5- to 6-fold) anti-cancer effects than each drug alone.

**Figure 6:**
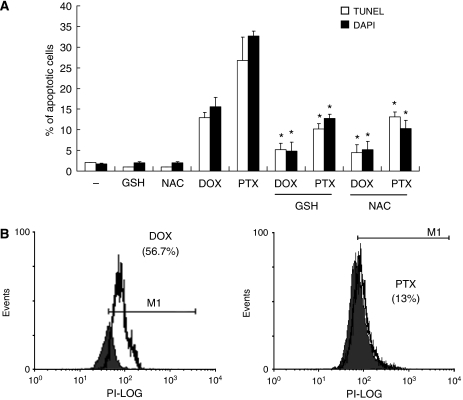
Involvement of ROS production in anti-cancer drug-induced apoptosis in ovarian cancer cells. SKOV-3 cells transfected with manganese superoxide disumutase (MnSOD) small-interfering RNA (siRNA) were pre-treated with glutathione (GSH, 5 mM) or *N*-acetyl cysteine (NAC, 5 mM) for 30 min, and then the cells were treated with doxorubicin (DOX, 5 *μ*M) or paclitaxel (PTX, 0.1 *μ*M) for another 48 h. (**A**) Apoptosis was measured by terminal deoxynucleotidyl transferase-mediated dUTP nick-end labelling (TUNEL) assay and 4′,6-diamidino-2-phenylindole (DAPI) staining. Experiments were repeated three times, and data are shown as mean±s.d. ^*^P<0.05 *vs* untreated control cells. (**B**) O_2_^•−^ level of DOX- and PTX-treated cells was measured by flow cytometry analysis using dihydroethidium. Solid histogram, untreated cells; open histogram, DOX- and PTX-treated cells.

Treatment of cells with antioxidants, such as GSH (5 mM) or NAC (5 mM), which can scavenge mitochondria-derived ROS, abolished the effects of MnSOD siRNA on DOX- and PTX-induced apoptosis (Figure 6A). Moreover, intracellular O_2_^•−^ was actually generated by the treatment of OVCAR-3 (data not shown) and SKOV-3 (Figure 6B) cells with each anti-cancer drug, suggesting a role for ROS in the process.

**Figure 7:**
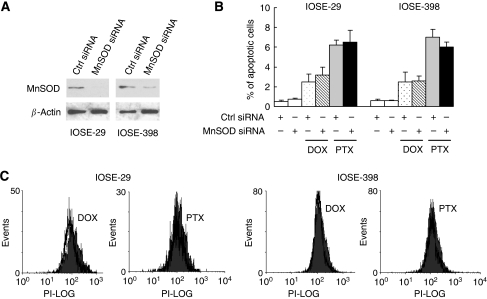
Inhibition of manganese superoxide disumutase (MnSOD) does not sensitise chemotherapy-induced apoptosis in non-transformed OSE cells. IOSE-29 and IOSE-398 cells were transfected with non-specific control (Ctrl small-interfering RNA, siRNA) or MnSOD siRNA in the presence or absence of doxorubicin (DOX, 5 *μ*M) or paclitaxel (PTX, 0.1 *μ*M) for 48 h. (**A**) Expression of MnSOD protein was analysed by western blotting. *β*-Actin serves as a protein-loading control. (**B**) Apoptosis was assessed by the terminal deoxynucleotidyl transferase-mediated dUTP nick-end labelling (TUNEL) assay. Experiments were repeated three times, and data are shown as mean±s.d. (**C**) O_2_^•−^ level of DOX- and PTX-treated cells was measured by flow cytometry analysis using dihydroethidium. Solid histogram, untreated cells; open histogram, DOX- and PTX-treated cells.

We also studied the combined effect of MnSOD siRNA and chemotherapeutic drugs in non-transformed OSE cells and did not find any synergistic effect. Figure 7A shows that a combination treatment of MnSOD siRNA with DOX and PTX induced 2.2 and 6.5% apoptosis respectively in OSE, which is almost the same as that by DOX or PTX alone. OSE showed only little, if any, increase of O_2_^•−^ in response to DOX and PTX treatment (Figure 7B) than did ovarian cancer cells (Figure 6B). Taken together, the above results suggest that the active generation of O_2_^•−^ in ovarian cancer cells by MnSOD inhibition renders these cells more susceptible to apoptosis induced by chemotherapeutic drugs.

### ERK1/2 is critical for MnSOD siRNA-mediated chemosensitisation

**Figure 8:**
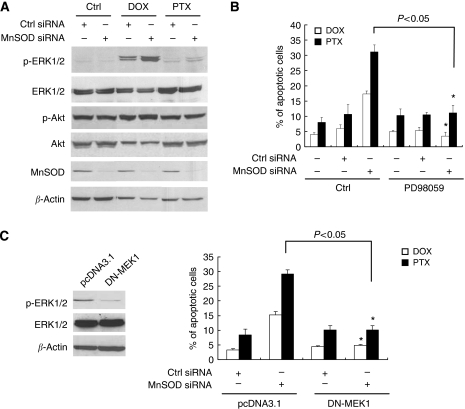
Inhibition of extracellular signal-regulated kinase (ERK)1/2 abolishes chemotherapy-induced apoptosis in manganese superoxide disumutase (MnSOD) small-interfering RNA (siRNA)-transfected SKOV-3 cells. (**A**) Cells transfected with non-specific control (Ctrl siRNA) or MnSOD siRNA in the presence or absence of doxorubicin (DOX, 5 *μ*M) or paclitaxel (PTX, 0.1 *μ*M) were analysed for activities of ERK1/2 and Akt by western blotting using antibodies specific for phosphorylated, activated forms of ERK1/2 and Akt. The membrane was then re-probed with total ERK1/2, Akt, and *β*-actin. Small-interfering RNA-mediated depletion of MnSOD was confirmed by western blot analysis using antibodies specific against MnSOD. (**B**) Cells were transiently transfected with either the Ctrl or MnSOD siRNA for 48 h, and then pre-treated with the ERK1/2 inhibitor (PD98059, 50 *μ*M) for 30 min followed by DOX or PTX addition for another 48 h. Cells were then harvested for terminal deoxynucleotidyl transferase-mediated dUTP nick-end labelling (TUNEL) assay. (**C**) Cells were transiently transfected with the control vector (pcDNA3.1) or DN-MEK1 for 24 h, and then treated with DOX or PTX for another 48 h. Whole-cell lysates were analysed for levels of phosphorylated and total forms of ERK1/2 by western blotting. *β*-Actin serves as a protein-loading control. Right, apoptosis was assessed by the TUNEL assay. Experiments were repeated three times, and data are shown as mean±s.d. ^*^P<0.05 *vs* untreated MnSOD siRNA-transfected cells as indicated.

Because phosphatidylinositol 3-kinase (PI3K) and mitogen-activated protein kinase (MAPK) are important in the regulation of survival in human ovarian cancer cells (Mills *et al*, 2001; Choi *et al*, 2003), we tested whether PI3K and MAPK are involved in MnSOD siRNA-mediated chemosensitisation. To this end, we first examined the activation of Akt and ERK1/2 in MnSOD siRNA-transfected and control cells by western blotting. As shown in Figure 8A, MnSOD had no effect on the phosphorylation of Akt. On the other hand, western blot analysis revealed significant levels of ERK1/2 phosphorylation in MnSOD siRNA-transfected but not in the Ctrl siRNA-transfected cells treated with DOX and PTX. In contrast to ERK1/2, MnSOD had no effect on p38 MAPK and JNK activation (data not shown).

We further explored whether ERK1/2 activation is involved in the MnSOD siRNA-mediated sensitivity to apoptosis in response to chemotherapeutic agents. To address this, SKOV-3 cells were pre-treated with 50 *μ*M of PD98059 for 30 min, and this was followed by treatment with DOX or PTX for a further 48 h. Figure 8B shows that the PD98059 abolished DOX- and PTX-induced apoptotic cell death in MnSOD siRNA-transfected cells (*P*<0.05 *vs* PD98059 untreated control cells). We further transfected DN-MEK1 vector into siRNA-transfected cells to examine their susceptibility to DOX and PTX. Again, the reduction in ERK1/2 activation by transfection with DN-MEK1 greatly suppressed DOX- and PTX-induced apoptosis in MnSOD siRNA-transfected cells but not the Ctrl siRNA-transfected cells as demonstrated by the TUNEL assay (Figure 8C; *P*<0.05 *vs* untransfected control cells). These results clearly demonstrate that MnSOD siRNA potentiates DOX- and PTX-induced apoptosis through the activation of ERK1/2.

### Caspase-9 acts as a downstream effector of ERK1/2

**Figure 9:**
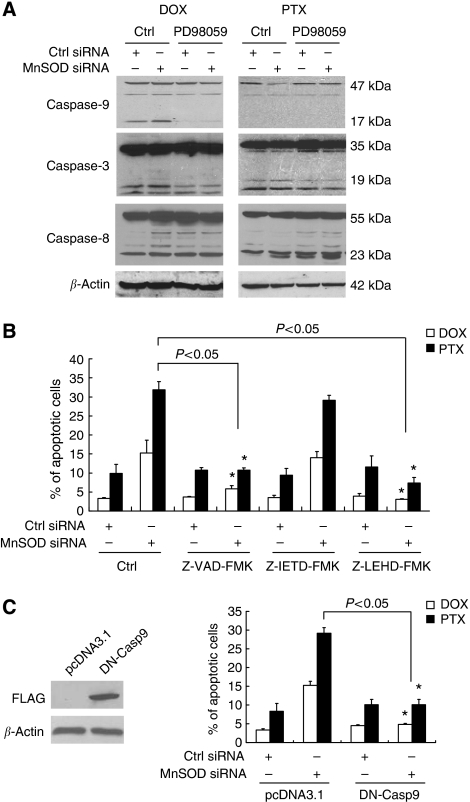
Manganese superoxide disumutase (MnSOD) small-interfering RNA (siRNA)-augmented apoptosis is involved in the activation of caspases. (**A**) SKOV-3 cells were transiently transfected with either the Ctrl or MnSOD siRNA for 48 h, and then pre-treated with the extracellular signal-regulated kinase (ERK)1/2 inhibitor (PD98059, 50 *μ*M) for 30 min followed by doxorubicin (DOX, 5 *μ*M) or paclitaxel (PTX, 0.1 *μ*M) addition for another 48 h. Cells were then analysed for expression of caspase-9, caspase-3, and caspase-8 proteins by western blotting using the respective antibodies. *β*-Actin serves as a protein-loading control. (**B**) Cells were transiently transfected with either the Ctrl or MnSOD siRNA for 48 h, and then pre-treated with the pan-caspase inhibitor (Z-VAD-FMK, 50 *μ*M), caspase-8 inhibitor (Z-IETD-FMK, 10 *μ*M), or caspase-9 inhibitor (Z-LEHD-FMK, 50 *μ*M) for 3 h, followed by DOX or PTX addition for another 48 h. Cells were then harvested for terminal deoxynucleotidyl transferase-mediated dUTP nick-end labelling (TUNEL) assay. Experiments were repeated three times, and data are shown as mean±s.d. ^*^P<0.05 *vs* untreated control cells. (**C**) Cells were transiently transfected with the control vector (pcDNA3.1) or FLAG-tagged dominant-negative mutant of caspase-9 (DN-Casp9) for 24 h, and then treated with DOX or PTX for another 48 h. Whole-cell lysates were analysed for expression of DN-Casp9 by western blotting using FLAG-specific antibodies. *β*-Actin serves as a protein-loading control. Right, apoptosis was assessed by the TUNEL assay. Experiments were repeated three times, and data are shown as mean±s.d. ^*^P<0.05 *vs* untreated MnSOD siRNA-transfected cells as indicated.

Caspases are major mediators of apoptosis (Lowe and Lin, 2000) whose activation depends on proteolytic cleavage of the procaspase to a smaller, enzymatically active form. To determine the mechanism underlying the MnSOD siRNA-mediated ERK1/2-dependent apoptotic effect, we examined the role of caspases by western blotting using antibodies that recognise both the procaspases and the cleaved subunits. As shown in Figure 9A, DOX treatment displayed a significantly enhanced cleavage of caspase-9, but not caspase-8, in cells transfected with MnSOD siRNA compared with cells transfected with control oligonucleotides. In addition, caspase-3 level was higher in MnSOD siRNA-transfected cells than in the Ctrl siRNA-transfected cells, suggesting its activation downstream of caspase-9. These effects were erased by the inhibition of MEK1 using PD98059. In both untreated and PTX-treated cells, although we failed to detect cleavage of caspase-9, procaspase-9 levels was lower in the MnSOD siRNA transfectants. Furthermore, addition of PD98059 prevented the processing of procaspase-9 to its active species (Figure 9B). These results indicate a possible role for the mitochondria apoptotic pathway rather than the death receptor pathway in chemosensitisation of DOX- and PTX-induced apoptosis in ovarian carcinoma cells.

**Figure 10:**
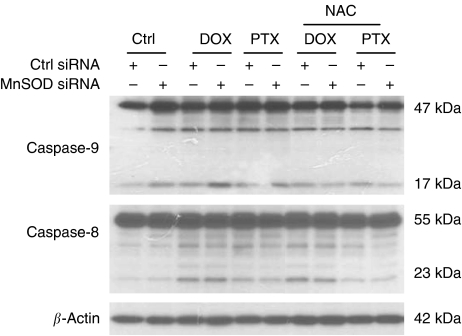
Involvement of ROS production in the manganese superoxide disumutase (MnSOD) small-interfering RNA (siRNA)-mediated activation of caspase-9. SKOV-3 cells were transiently transfected with either the Ctrl or MnSOD siRNA for 48 h, and then pre-treated with *N*-acetyl cysteine (NAC, 5 mM) for 30 min followed by doxorubicin (DOX, 5 *μ*M) or paclitaxel (PTX, 0.1 *μ*M) addition for another 48 h. Cells were then analysed for expression of caspase-9 and caspase-8 proteins by western blotting using the respective antibodies. *β*-Actin serves as a protein-loading control.

To ascertain the role of caspase-9, we treated siRNA-transfected cells with a specific inhibitor of caspase-9 (Z-LEHD-FMK) and then examined their sensitivity to apoptosis. Under these conditions, the DOX- or PTX-induced apoptosis was clearly prevented by the caspase-9 inhibitor Z-LEHD-FMK (Figure 9B). Similarly, treatment the cells with the pan-caspase inhibitor (Z-VAD-FMK) totally inhibited DOX- or PTX-induced apoptosis. In contrast, the inhibitor of caspase-8 (Z-IETD-FMK) had no effect on apoptosis. To confirm these data, a dominant-negative mutant of caspase-9 was used, and it also effectively inhibited apoptotic response to DOX and PTX in MnSOD-depleted cells (Figure 9C). Furthermore, pre-treatment of GSH (data not shown) or its precursor NAC (Figure 10) prevented the activation of caspase-9 caused by MnSOD siRNA. Consistent with the results described above, there was no effect on caspase-8 (Figure 10). These data suggest that cellular oxidative status could affect MnSOD siRNA-induced apoptosis to DOX or PTX by regulating caspase, the central component of the apoptotic pathways, and in particular caspase-9 activation.

## Discussion

DOX and PTX are two of the most widely used anti-cancer drugs in the clinical treatment of ovarian cancer. In addition to the dose-limiting toxicity, these two agents induce drug resistance, which still pose a major problem for their clinical use (Agarwal and Kaye, 2003). In the present study, we demonstrate that MnSOD siRNA specifically exerts an enhancing role on ovarian cancer cell apoptosis induced by chemotherapeutic treatment and that this effect is restricted to the activation of intrinsic apoptotic pathways through ERK1/2. Importantly, these changes in chemotherapy sensitivity occurred at pharmacologically attainable drug levels (Karlsson *et al*, 1999). Moreover, low concentrations of DOX and PTX with MnSOD siRNA can also induce an enhanced cytotoxic response in the cancer cells (data not shown), suggesting potential utility to improve clinical efficacy.

These intriguing findings are relevant to a large number of ovarian carcinomas. We and others have observed that ovarian carcinomas constitutively overexpress MnSOD compared with normal cells and tissues, and increased MnSOD has been detected in ∼70% of malignant ovarian cancer tissues (Ishikawa *et al*, 1990; Hileman *et al*, 2004; Hu *et al*, 2005), although there was some variations among the individual samples. This variation likely reflects the various degrees of ROS stress and other yet unknown changes in the respective cell lines or cancer tissues. Therapeutic selectivity is one of the most important considerations in cancer treatment. The differential expression of MnSOD in ovarian cancer and normal cells likely is important in mediating the cancer-selective effects that we observed. Although the precise underlying mechanisms responsible for the increase of MnSOD expression in ovarian cancer remain unclear at the present time, MnSOD has been shown to be inducible by multiple factors such as hypoxia, ROS, and inflammatory cytokines including interleukin-1 and tumour necrosis factor (Masuda *et al*, 1988; Wong and Goeddel, 1988). MnSOD has also been identified as a potential target for the tumour suppressor protein p53 (Pani *et al*, 2000), which is frequently mutated in a variety of human cancers, including those of the ovary. Moreover, it has been known for a long time that due to the Warburg effect, cancer cells are under intrinsic oxidative stress that likely forces these cells to rely more on antioxidant enzymes such as MnSOD for O_2_^•−^ elimination, thus making the malignant cells more vulnerable to MnSOD inhibition than normal cells (Pelicano *et al*, 2004). Supporting this view, high expression of MnSOD and other antioxidant enzymes such as Cu/Zn-SOD was observed in ovarian cancer cells (Supplementary Figure 2; Hu *et al*, 2005). We also showed that the ovarian cancer cells were more sensitive to DOX and PTX than non-transformed OSE by MnSOD inhibition, as demonstrated by the significant accumulation of O_2_^•−^ and the subsequent apoptosis.

One mechanism that could underlie the enhancing effect of MnSOD siRNA on the cytotoxic chemotherapeutic agents is the reduction in the cellular capacity to withstand the oxidative damage exerted by these agents. The generation of O_2_^•−^ radicals appears to be a critical event in mediating drug-induced apoptosis, because both DOX and PTX were able to induce O_2_^•−^ generation in human ovarian cancer cells and inhibition of ROS generation by NAC or GSH effectively protected the cells from the cytotoxic effects. The reduction in the cellular activity of the constitutive enzyme MnSOD to suppress O_2_^•−^ elimination could also underlie the potentiation of DOX and PTX cytotoxicity. It is worthy to note here that the MnSOD-expressing SKOV-3 is more resistant to standard chemotherapy than MnSOD low OVCAR-3 cells (Figures 3B and 5B; Gibb *et al*, 1997). When accumulated to high levels, ROS are chemically reactive and toxic to the cells. Given that ROS stress is dependant on the balance between O_2_^•−^ generation and elimination, interference of both processes is expected to cause a severe oxidative stress and may enhance the killing of cancer cells.

The signal transduction mechanism by which MnSOD functions in cell survival or apoptosis of any specific cell type is not clearly defined. We showed that apoptosis enhancement by MnSOD siRNA was mediated through the activation of an MEK1-ERK1/2 signalling pathway. The role of ERK1/2 in drug resistance has been extensively exploited in different cell systems. It has been shown to be a survival signalling factor. Here, in contrast, we find that the activation of ERK1/2 is associated with cancer cell apoptosis. The MEK1 inhibitor PD98059 suppressed ERK1/2 activation and effectively protected the MnSOD siRNA-transfected cells from the drug-induced apoptosis. This observation however seems to be consistent with the mechanism of drug-resistant action in ovarian cancer. Several studies have demonstrated that ERK1/2 activity is elevated in human ovarian tumours, and its activation conferred resistance to chemotherapeutic agents (Pan *et al*, 2002; Steinmetz *et al*, 2004; Lee *et al*, 2007). Cisplatin, another platinum-based chemotherapy, has also been found recently to enhance ovarian cancer cell resistance to apoptosis through activation of ERK1/2 (Lee *et al*, 2007). Other members of the MAPK family, such as p38 MAPK and JNK, are not involved. It has also been postulated that MnSOD via O_2_^•−^ elimination to regulate cell proliferation and tumourigenesis through the activation of Ras-mediated signalling pathway (Yang *et al*, 1999; Ridnour *et al*, 2004). How ERK1/2 mediates cellular apoptosis remains poorly understood, but it appears that this may occur at different levels (Zhuang and Schnellmann, 2006). Extracellular signal-regulated kinase 1/2 may act upstream of mitochondrial cytochrome *c* release and caspase-3 activation through upregulation of Bax and p53. Alternatively, ERK1/2 can regulate the apoptotic pathway at the level of caspase-8. There is also evidence that ERK1/2 can promote apoptosis through suppression of Akt-mediated survival signal. However, although PI3K/Akt is important in ovarian cancer biology and tumourigenesis (Yuan *et al*, 2000; Mills *et al*, 2001), our results suggest that it is not involved in the apoptosis induced by MnSOD siRNA in combination with DOX and PTX.

There are two major cellular pathways of drug-induced apoptosis: the mitochondrial pathway, initiated by release of cytochrome *c*, and the death receptor pathway, initiated by ligation of the Fas receptor by its ligand FasL (Green, 1998). Previously, Fujimura *et al* (1999) reported that MnSOD inhibits release of cytochrome *c* to the cytosol and reduces apoptosis after permanent focal cerebral ischaemia. Here we showed that MnSOD siRNA increased caspase-9 activation and drug-induced apoptosis through an MEK1-ERK1/2-dependent mechanism, thereby enhancing the ability of chemotherapeutic agents to kill tumour cells. The caspase-8/death receptor pathway of apoptosis is shown to be activated by chemotherapy-induced apoptotic signals such as ROS (Xia *et al*, 2005). Evidence is also emerging that death receptor function may be involved in the control of ovarian cancer cell apoptosis (Wajant, 2003). However, in this study, we ruled out this possibility because MnSOD inhibition had no effect on caspase-8 activity. Nor did treatment of cells with a caspase-8 inhibitor (Z-IETD-FMK) affect apoptosis.

In conclusion, this study reveals important mechanism-based knowledge of the synergism between MnSOD inhibition and clinically relevant anti-cancer agents. These findings warrant further consideration as a novel strategy of treating refractory tumours to convey survival benefits for ovarian cancer patients. Our results also suggest that higher levels of MnSOD in ovarian cancer may be a good prognostic marker for chemotherapy.
