# Supplementary figures and images for: Chemosensitisation by manganese superoxide dismutase inhibition is caspase-9 dependent and involves extracellular signal-regulated kinase 1/2
Source: Br J Cancer. 2008 Jul 1;99(2):283–93. doi: 10.1038/sj.bjc.6604477 (PMC2480972; doi:10.1038/sj.bjc.6604477)

Supplementary Figure 1

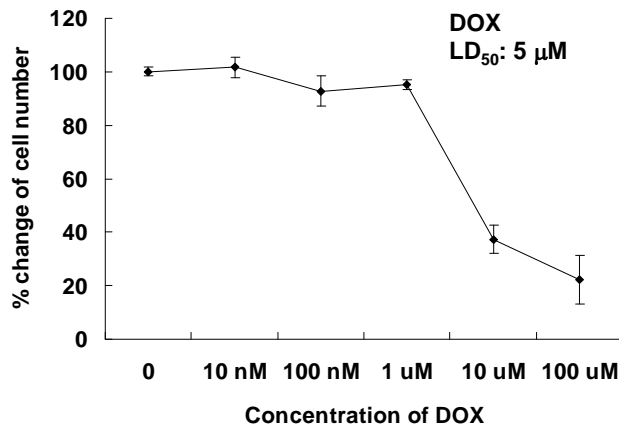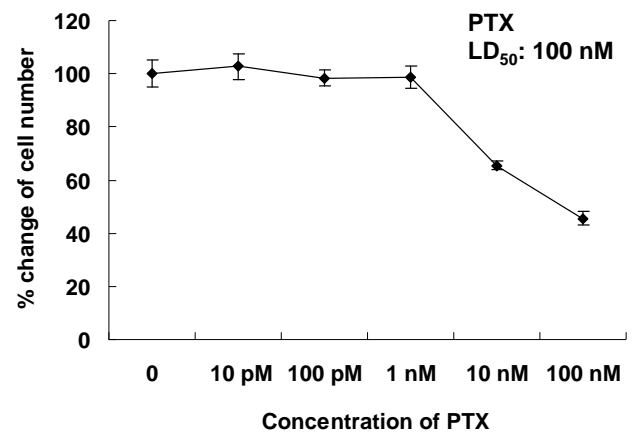

Supplementary Figure 2

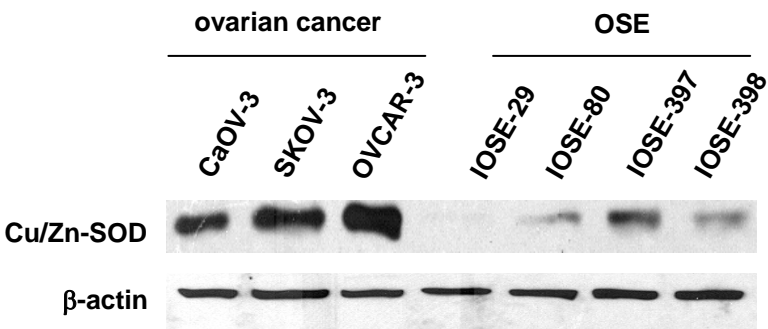

Supplement: Supplementary Figures [file 6604477x1.pdf]
