# Supplementary material for: Chemosensitisation by manganese superoxide dismutase inhibition is caspase-9 dependent and involves extracellular signal-regulated kinase 1/2
Source: Br J Cancer. 2008 Jul 1;99(2):283–93. doi: 10.1038/sj.bjc.6604477 (PMC2480972; doi:10.1038/sj.bjc.6604477)
Supplement: Supplementary Figure Legends [file 6604477x2.doc]

**Supplementary Figure Legends**

**Supplementary Figure 1** LD_50_ for chemotherapeutic drugs in OVCAR-3. Equal number of cells was treated with increasing concentrations of doxorubicin (DOX) or paclitaxel (PTX) indicated for 48 h, and then harvested for MTT assays. Absorbance reading from the untreated control was set as 100%. Experiments were repeated three times in triplicates, and data are shown as mean ± SD.

**Supplementary Figure 2** Cu/Zn-SOD expression in OSE and ovarian cancer cells. Whole cell lysates from different ovarian cell lines containing equal amounts of protein (50 μg) were loaded in each lane and analyzed for expression of Cu/Zn-SOD by Western blotting. β-actin was also blotted as a protein loading control.
